# Synthesis of Gold Nanoparticles Using Leaf Extract of *Ziziphus zizyphus* and their Antimicrobial Activity

**DOI:** 10.3390/nano8030174

**Published:** 2018-03-19

**Authors:** Alaa A. A. Aljabali, Yazan Akkam, Mazhar Salim Al Zoubi, Khalid M. Al-Batayneh, Bahaa Al-Trad, Osama Abo Alrob, Alaaldin M. Alkilany, Mourad Benamara, David J. Evans

**Affiliations:** 1 Faculty of Pharmacy, Yarmouk University, P.O.BOX 566, Irbid 21163, Jordan; yazan.a@yu.edu.jo (Y.A.); osama.yousef@yu.edu.jo (O.A.A.); 2 Department of Basic Medical Sciences, Faculty of Medicine, Yarmouk University, Irbid 21163, Jordan; mszoubi@yu.edu.jo; 3 Department of Biological Science, Yarmouk University, P.O.BOX 566, Irbid 21163, Jordan; albatynehk@yu.edu.jo (K.M.A.-B.); bahaa.tr@yu.edu.jo (B.A.-T.); 4 School of Pharmacy, University of Jordan, Aljubeiha, Amman, Jordan 11942, Jordan; a.alkilany@ju.edu.jo; 5 Institute for Nanoscience, University of Arkansas, Fayetteville, AR 72701, USA; mourad@uark.edu; 6 John Innes Centre, Norwich Research Park, Norwich NR4 7UH, UK; Dave.Evans@jic.ac.uk

**Keywords:** biosynthesis, gold nanoparticles, *Ziziphus zizyphus*, antifungal activity, green chemistry, nanomaterials, biopharmaceutics

## Abstract

(1) Background: There is a growing need for the development of new methods for the synthesis of nanoparticles. The interest in such particles has raised concerns about the environmental safety of their production methods; (2) Objectives: The current methods of nanoparticle production are often expensive and employ chemicals that are potentially harmful to the environment, which calls for the development of “greener” protocols. Herein we describe the synthesis of gold nanoparticles (AuNPs) using plant extracts, which offers an alternative, efficient, inexpensive, and environmentally friendly method to produce well-defined geometries of nanoparticles; (3) Methods: The phytochemicals present in the aqueous leaf extract acted as an effective reducing agent. The generated AuNPs were characterized by Transmission electron microscopy (TEM), Scanning electron microscope (SEM), and Atomic Force microscopy (AFM), X-ray diffraction (XRD), UV-visible spectroscopy, energy dispersive X-ray (EDX), and thermogravimetric analyses (TGA); (4) Results and Conclusions: The prepared nanoparticles were found to be biocompatible and exhibited no antimicrobial or antifungal effect, deeming the particles safe for various applications in nanomedicine. TGA analysis revealed that biomolecules, which were present in the plant extract, capped the nanoparticles and acted as stabilizing agents.

## 1. Introduction

Recently, metallic nanoparticles have received much attention because of their distinctive optical, magnetic, and catalytic properties. The size, shape, monodispersity, and morphology of the particles are essential to tune these properties [1]. Various synthesis methods have been developed to formulate such nanoparticles, including chemical, physical, and biological methods [2,3,4]. 

The typical chemical synthesis of metal nanoparticles can lead to the production of toxic compounds, which remain adsorbed on the particle surface and have adverse effects on human health. For example, the highly toxic quaternary ammonium surfactant cetyltrimethylammonium bromide is still the “magic salt” used to prepare gold nanorods. However, the green synthesis of nanoparticles offers an alternative route utilizing the natural ingredients present in plant extracts from, for example, coriander, *Bischofia javanica* (L.), *Daucus carota*, *Solanum lycopersicums*, *Hibiscus*, *cannabinus* leaf, lemongrass, *Moringa oliefera* flower, *Bacopa monnieri*, *Citrus unshiu* peel, lemongrass (*Cymbopogon flexuosus*) [5], the plant extract of *Aloe Vera* leaves [6], and *Ananas comosus* [7,8,9,10,11,12]. There are several other published reviews reporting the green synthesis of different types of nanoparticles from plant extracts [13,14,15,16,17,18,19,20,21,22]. 

Gold nanoparticles (AuNPs) are a promising class of nanomaterials with many varieties of applications, which includes cancer hyperthermia treatment [23], surface-enhanced Raman spectroscopy (SERS) [24], and infrared radiation absorbing optics [25]. Consequently, a variety of synthetic procedures for the formation of various shapes and sizes of AuNPs have been reported [26]. Several isotropic shapes including rods, wires, plates, and teardrop structures can be obtained by wet chemical synthesis routes [27]. Conversely, biosynthetic methods that use biological microorganisms, plants, or plant extracts have emerged as simple and eco-friendly “green” alternatives to chemical approaches. 

It has been reported that inorganic nanoparticles can interact with microorganisms and consequently may exhibit antibacterial and antifungal activity [28,29,30,31]. A few reports have also shown that gold nanoparticles exhibit significant antimicrobial activity [32,33]. Their prevalent antimicrobial activity may be credited to their strong cytotoxicity to varied microorganisms; the interaction with various surface-exposed functional groups present on the bacterial cell surface may lead to bacterium destruction and inactivation. This activity has been attributed to either a coating on the AuNPs surface or to reaction contaminants left over from the manufacturing approach rather than the AuNPs cores [34]. There are two main opinions about the toxicity of nanoparticles in the literature: on one hand, some report that AuNPs are nontoxic regardless of their size (3.5, 4, 10, 12, or 18 nm) or capping agents such as citrate, cysteine, biotin, sugars, etc. [35,36,37]. On the other hand, others reported that the toxicity of 2 nm cationic AuNPs is dose-dependent. However, the same nanoparticles with a negative surface charge were deemed nontoxic at the same concentrations [35,38].

*Ziziphus zizyphus* is a spiny shrub (known locally as “Ennab”) that is cultivated in the Middle East (including Hashemite Kingdom of Jordan) and North Africa. Ennab flowers are small and yellow-green in color, bearing sweet brown fruits when ripe that are often consumed as snacks by the locals [39,40]. It has been reported that traditional medicines of the Chinese and Korean uses the Ennab fruit and its seeds to alleviate stress [41,42]. Furthermore, Ennab fruits have been reported to have antifungal, antibacterial, antiulcer, and anti-inflammatory activities [43,44,45].

Here, we demonstrate the synthesis of a monodisperse AuNPs, with various geometries, by a very simple single-step synthesis at ambient temperature through the reduction of aqueous chloroaurate ions (AuCl_4_^−^) with the aqueous extract of the Ennab leaves. The prepared AuNPs were characterized using transmission electron microscopy (TEM), scanning electron microscopy (SEM), atomic force microscopy (AFM), UV-vis spectroscopy, and energy dispersive X-ray spectroscopy (EDX). Further, the antibacterial and antifungal properties of the AuNPs against Gram-negative bacteria (*E. coli*) and yeast (*Candida albicans*) were investigated.

## 2. Materials and Methods 

### 2.1. Materials 

Tetrachloroauric acid (HAuCl_4_·3H_2_O) was obtained from Sigma-Aldrich (Saint Louis, MO, USA). An aqueous solution of HAuCl_4_ (1 mM) freshly prepared in double-distilled (DD) water was used throughout the experimental work reported here. All glassware used in this work was thoroughly rinsed with pure water before starting.

### 2.2. Methods

#### 2.2.1. Gold Nanoparticles Synthesis 

The plant extract for the reduction of Au^3+^ ions to Au^0^ was prepared by combining thoroughly washed Ennab leaves (10 g; leaves were collected in the month of June) in a 200 mL Erlenmeyer flask with sterile DD water (100 mL). The mixture was then boiled for 5 min. In a typical experiment, 5 mL of the plant extract was added to 1 mM aqueous HAuCl_4_ solution (45 mL). Reduction of AuCl_4_^−^ was monitored by recording the UV-vis absorption spectrum as a function of time. 

#### 2.2.2. Purification of AuNPs

After the completion of the reaction, AuNPs were spun at 14,000 RPM (bench top, Eppendorf, Thermo Fisher Scientific, Darmstadt, Germany) for 20 min at ambient temperature to eliminate any large aggregates, the supernatant was collected and further purified on PD-10 columns (GE Healthcare, Chicago, IL, USA), and eluted samples (3.5 mL in total) were collected and dialyzed against 10 mM sodium phosphate buffer with a pH of 7.0 using 20 kDa dialysis bags (Spectrum Labs) with buffer exchange after 2 h, followed by overnight incubation for 15–18 h.

#### 2.2.3. UV-visible Spectroscopy

To determine the optimum concentration of plant extracts, UV-vis was used at different time points while fixing the concentration of the plant extract and the aqueous solution of gold chloride. The visual indication of the color exchange and the formation of ruby-red color indicated the formation of the AuNPs. The formation of the AuNPs was confirmed by scanning the absorption maxima of the AuNPs colloid between 200 and 800 nm on a PerkinElmer Lambda 25 spectrometer (PerkinElmer, Buckinghamshire, UK). The color change was observed 0.5 min after the mixing of the plant extract and gold chloride solution. The nanoparticle formation was completed within 3 min of the reaction initiation. The spectroscopic analyses were carried out on a freshly prepared sample at ambient room temperature (24–28 °C) using quartz cuvettes with an optical path length of 1 cm.

#### 2.2.4. X-ray Diffraction (XRD)

X-ray diffraction measurements were taken on a MAXima_X XRD-7000 (Shimadzu, Tokyo, Janpan operating at a voltage of 40 kV and a 20 mA electrical current with a Cu-Kα (λ = 1.54 Å) radiation source in the region of 2θ from 30° to 75°. Colloidal AuNPs were centrifuged at 10,000× *g* for 15 min at ambient temperature. Pellets were washed with DD water three times with 5 mL each, and the sample was freeze-dried (−54 °C under vacuum and pressure) prior to the analysis. 

#### 2.2.5. Thermogravimetric Analysis (TGA)

TGA was performed using a PerkinElmer Diamond TG/DTA STA 6000 (PerkinElmer, Buckinghamshire, UK) operating between room temperature and 900 °C at a heating rate of 10 °C·min^−1^ with an O_2_ flow of 20 mL·min^−1^. The freeze-dried sample (−54 °C under vacuum and pressure) was loaded to a clean pan supported by a precision balance. The mass of the dried sample was monitored and recorded at the beginning and during the experiment. The sample temperature was raised to 100 °C and held at that temperature for 15 min to ensure moisture removal from the sample before allowing the set temperature to increase gradually according to the set rate. 

#### 2.2.6. Transmission Electron Microscopy (TEM) 

TEM was performed using an FEI Titan Transmission Electron Microscope FEI company, Hillsboro, OR, USA) operating at 300 kV and fitted with a post-column Gatan Tridiem GIF 863 Microscope (Gatan, Pleasanton, CA, USA). The samples were first dispersed in water at a concentration of 0.05 mg/mL, then deposited on Lacey carbon grids, 300 mesh (SPI supplies, 3330C-CF) and air dried prior to imaging.

#### 2.2.7. Scanning Electron Microscopy (SEM) and Energy-Dispersive X-ray Spectroscopy (EDX)

The morphology and the geometry of the AuNPs were investigated by an FEI Nova Nanolab 200 scanning electron microscope (FEI company Hillsboro, OR, USA). The elemental composition of the nanoparticles colloid was determined using energy dispersive X-ray spectroscopy using a Bruker X-flash detector (Bruker, Bremen, Germany). The energy of the electron beam was kept at 15 keV for both imaging and EDX analysis.

#### 2.2.8. Atomic Force Microscopy (AFM)

Diluted samples (0.05 mg/mL in water) were spread on a zinc substrate for examination by AFM. The topography of the sample from a scanned area of 1× 1 µm was evaluated for a set point of 10 nm and a scan rate of 1 µm/s. The images were analyzed using a Bruker Dimension 3100 with Nanoscope 5 software (Bruker, Bremen, Germany). 

#### 2.2.9. Dynamic Light Scattering and Zeta Potential

The hydrodynamic diameter of gold nanoparticles was determined using a Zeta-PAL (zeta potential analyzer) (Brookhaven, NY, USA). All AuNPs samples (50 µg/mL) were suspended in deionized water. Ten runs with a 30 s duration each were set for each measurement. Each measurement was repeated three times under the following conditions: 25 °C, electric field 13.89 V/cm, refractive index 1.330, and voltage 5 V. The mean zeta potential was calculated using the Smoluchowski coagulation equation at a 659 nm wavelength with (seven) automatic attenuation settings. Data were reported from three independent syntheses; each set of measurements had 10 replicates.

#### 2.2.10. Antimicrobial Activity Assay

The antibacterial activity of AuNPs and gold ions was qualitatively determined by a radial diffusion assay using *E. coli* (ATCC number 25922) as a representative Gram-negative bacterium. The bacteria were grown from broth on nutrient agar. Wells with a disk size of 8 mm were generated using a standard punch. Fifty microliters AuNPs suspension or 50 µL mM aqueous HAuCl_4_ solution were added to either well followed by overnight incubation at 37 °C. The inhibition zones (mm) were recorded and the antimicrobial activities against *E. coli* was analyzed. 

#### 2.2.11. Fungicidal Activity Assays

Fungicidal activity was determined by microdilution plate assay using *C. albicans* (SC5314) as described previously [46]. Briefly, cell suspensions (20 µL of 1.8 × 10^5^ cells/mL, suspended in 20 mM sodium phosphate buffer at pH 7.4) were mixed with 20 µL of (5, 2.5, 1.25 mg/mL) AuNPs in water, and incubated for 2 h at 37 °C with shaking at a speed of 550 RPM. The reaction was diluted by the addition of 360 µL phosphate buffer (5 mM/pH 7), after which 40 µL of cell suspension was spread on Sabouraud dextrose agar and incubated for 24 h at 37 °C. Loss of viability was calculated as [1 − (colony-forming unit CFUs in the presence of AuNPs/CFUs with no particles)] × 100.

#### 2.2.12. Plate Spotting and Colony Counting

From an overnight culture, 50 µL of 1 × 10^4^ cells/mL yeast extract peptone dextrose agar (YPD) of strain SC5314 was mixed with 50 µL of AuNPs (5, 2.5, and 1.25 mg/mL water), and incubated overnight for 20 h at 30 °C with shaking at 170 RPM. Later, five serial dilutions (1:10) were made, and 4 µL was spotted onto a YPD plate. For colony counting, 30 µL from the last dilution (approximately 104 cells/mL) was plated on a YPD plate, incubated at 30 °C, and counted after 48 h. The positive control was cells without AuNPs. The negative control was buffer and AuNPs without cells (this was to test whether the nanomaterials contained any contaminants). Cell viability was then calculated relative to the control.

## 3. Results and Discussion 

### 3.1. Synthesis of AuNPs

The standard method of the synthesis of AuNPs with similar sizes to those reported in this work was achieved by Turkevich and Frensby through the reduction of gold hydrochlorate solution by sodium triscitrate solution at 100 °C [47]. Herein, we report the use of a simpler (one-step) and greener method for the synthesis of AuNPs. Furthermore, analysis of the prepared nanoparticles using ImageJ software (IF1.46r) for particle counting and distribution from TEM images revealed that approximately 90% of the imaged particles were spherical and monodisperse, which is a major advantage of this green synthesis. This can only be matched using harsh and expensive chemicals. 

Upon mixing the Ennab leaf extract with aqueous chloroauric acid, the solution transmuted color rapidly from pale yellow to vivid ruby-red, indicating the formation of AuNPs. AuNPs (with a diameter less than 30 nm) exhibit a visible ruby-red color due to the localized surface plasmon resonance (SPR) [48,49]. The accepted hypothetical mechanism for the synthesis of NPs in this way is by a phytochemical-driven reaction in which the plant extract contains complex reducing molecules such as antioxidants, enzymes, and phenolic moieties, which reduce gold cations into AuNPs [50,51,52,53]. The hypothetical reduction of HAuCL_4_ is driven by the presence of the phytochemicals to form zerovalent gold, which will subsequently lead to the agglomeration of gold atoms to nanosized particles, which are finally stabilized by the phytochemicals to give isotropic (spherical) AuNPs.

Photosynthetic plants, including Ennab, contain a complex biological network of antioxidant metabolites and enzymes that work collectively to prevent oxidative damage to cellular components [54]. Earlier publications show that plant extracts contain biomolecules including polyphenols, flavonoids, ascorbic acid, sterols, triterpenes, alkaloids, alcoholic compounds, saponins, β-phenylethylamines, polysaccharides, glucose, fructose, and proteins/enzymes, which could act as reductants for metal cations, leading to the formation of NPs [55]. It also seems probable that glucose and ascorbate can reduce silver and gold ions to form nanoparticles at elevated temperatures [56,57]. Proteins, enzymes, phenolics, and other chemical compounds within plant leaf extracts can reduce silver salts and provide exquisite tenacity toward the agglomeration of the formed nanoparticles [27,58,59]. In Neem leaf extract, terpenoids, polyphenols, sugars, alkaloids, phenolic acids, and proteins play crucial roles in the bio-reduction of metal ions, yielding nanoparticles [27].

The generated AuNPs exhibited excellent colloidal stability upon mixing with the used nutrient-rich medium. Incubating AuNPs with the nutrient media did not generate any visible aggregation nor change the color. The culture media contains amino acids and proteins that might act as stabilizing and surface capping agents to preserve colloidal stability in biological mediums [60]. 

### 3.2. Characterization of AuNPs

#### 3.2.1. UV-visible Spectroscopy 

**Figure 1:**
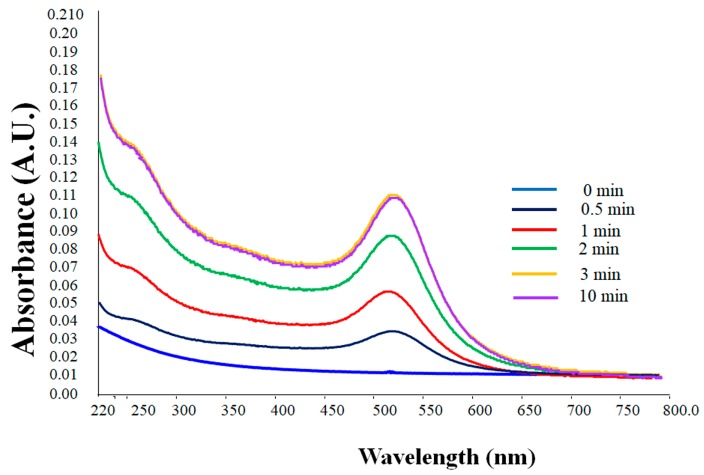
UV-vis absorption spectra of the formed gold nanoparticles (AuNPs) synthesized using Ennab leaf extract. The spectrum suggests that the complete reduction of gold ions using leaf extract was completed within 3 min after mixing the solutions.

The formation of AuNPs was evident from the change in solution color from light-yellow to ruby-red as well as from the presence of the typical plasmon peak in the range of 525–540 nm with a peak maximum in the range of approximately 527–535 nm in the UV-vis spectrum. The peak is a distinctive characteristic of spherical AuNPs with a diameter of 30–50 nm [61,62]. Monitoring the reaction kinetics using UV-vis spectroscopy confirmed the completion of the reaction after 3 min as evident from the stability of the plasmonic peak, with no significant change beyond this time, as shown in Figure 1. The concentration of the generated AuNPs was determined spectrophotometrically using the Beer-Lambert law with an extinction coefficient ɛ of 1.8 × 10^10^ M^−1^∙cm^−1^ for a particle diameter of 50 nm [62].

#### 3.2.2. Dynamic Light Scattering (DLS) and Zeta Potential (ζ)

**Figure 2:**
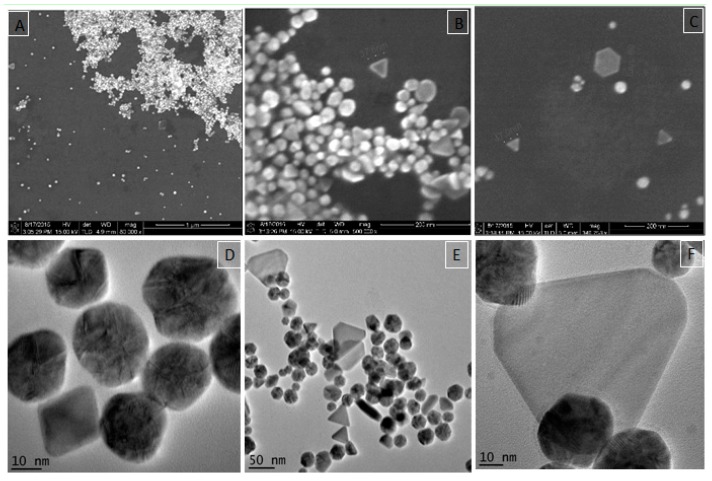
Unstained electron microscope images of AuNPs; SEM images (**A**–**C**) and bright-field TEM images (**D**–**F**). The shape of most NPs is spherical while triangular and hexagonal platelets, as well as truncated AuNPs, are also observed. The sample contains different lattice formations from single layer “Au” to multilayers (**D**,**F**). Scale bars in Figure 2A is 1 um, Figure 2B,C is 200 nm.

DLS analysis of the generated AuNPs showed an average hydrodynamic diameter of 51.8 ± 0.8 nm. The polydispersity index of the AuNPs was 0.340%, which is consistent with a ‘medium monodisperse’ distribution [63,64]. Medium monodispersity may arise from the size or shape heterogeneity. TEM images confirmed the presence of various geometries in the samples (Figure 2) that are dominated by spheres. 

Zeta potential values are often used as a hallmark indication of the stability of colloidal particles. The absolute values replicate the net electrical charge on the particles’ external surface that arises from the surface functional groups. Nanoparticles are considered to exist as stable colloids if their zeta potential is more than 25 mV or less than –25 mV [63,64]. The zeta potential of the AuNPs was −40.4 ± 0.2 mV; the suspension of AuNPs in a buffer formed a stable colloid (well-dispersed) with no visible aggregation over 6 months. 

#### 3.2.3. Electron Microscopy

**Figure 3:**
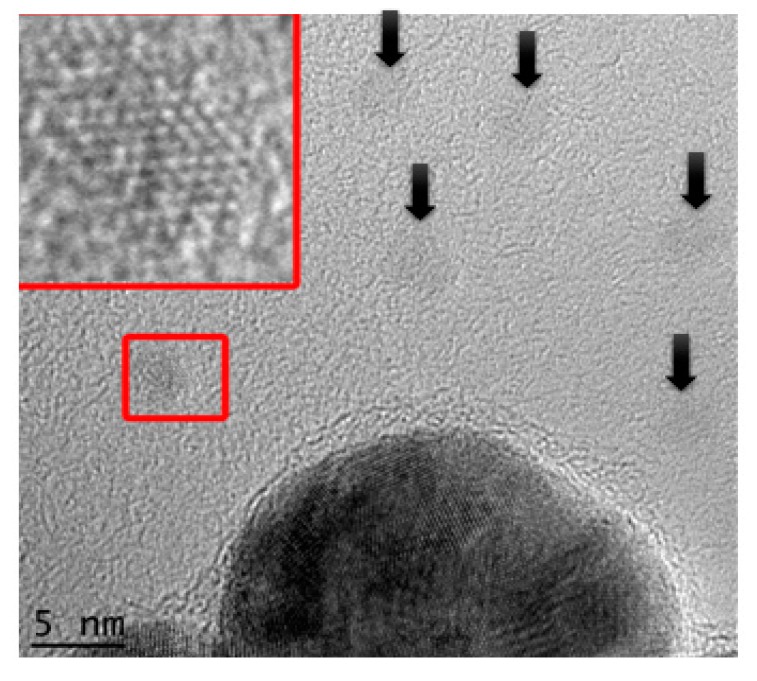
Unstained TEM image showing the presence of small AuNPs with an average diameter of 3.22 ± 0.5 nm (measured from the image). Arrows indicate single 3 nm particles. The inset image is a higher magnification graph of one of the AuNPs showing the gold lattice arrangement.

SEM and TEM images revealed that the generated particles mainly consist of spherical, poly-crystalline AuNPs. Interestingly, anisotropic shapes such as triangular and hexagonal platelets in addition to truncated single nanosheets appeared almost in all imaged samples (Figure 2). The truncation geometries appeared as a common feature in such disk-like nanostructures and has been reported for chemically synthesized AuNPs [65,66] and silver nano-triangles [67,68]. Image analysis using Image-J indicated that the overall percentage of gold triangular and hexagonal NPs were approximately 10% of the total population. In addition, a small amount of AuNPs with a size of 3 nm were also observed in some TEM images (Figure 3). TEM at higher magnification confirmed the lattice structure of these particles (Figure 3, inset). 

**Figure 4:**
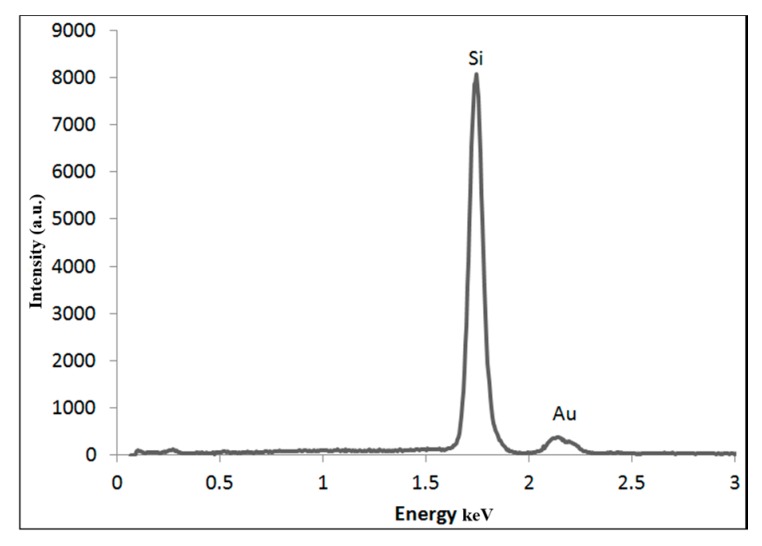
Energy dispersive X-ray (EDX) analysis confirms that AuNPs contain gold only. The Si signal is from the silicon substrate.

**Figure 5:**
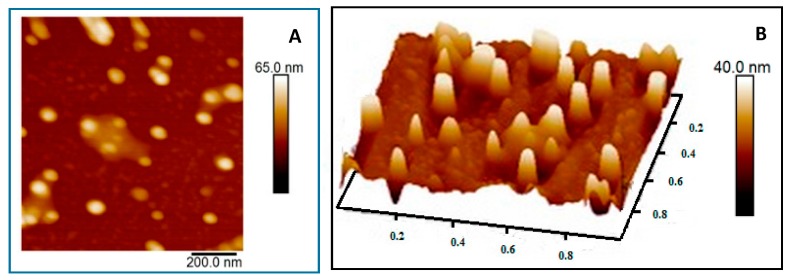
Atomic force microscopy (AFM) images showing the surface topology and size distribution of AuNPs in solution. (**A**) Two-dimensional (2D) image and (**B**) three-dimensional (3D) reconstructed image of the generated particles.

SEM-EDX confirmed that the NPs are primarily composed of gold (Figure 4). This finding excludes the presence of any contaminants. Furthermore, AFM analysis showed that the particles are monodisperse and with narrow size distribution as shown in (Figure 5).

#### 3.2.4. AFM

AFM analysis evaluated the presence and size distribution of the generated AuNPs. The scanning area was 1 × 1 µm in a tapping mode and both two-dimensional (2D) and three-dimensional (3D) images were generated (Figure 4). The images confirm the uniform distribution of AuNPs as most of the particles were approximately 40–50 nm in diameter with a sphere topology, consistent with the DLS and TEM measurements. 

#### 3.2.5. XRD Analysis

**Figure 6:**
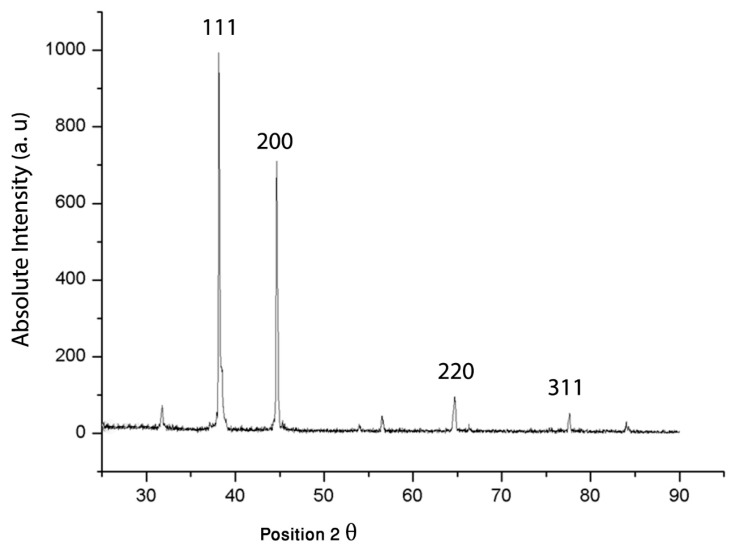
X-ray diffraction (XRD) of crystalline AuNPs characterized by the presence of four peaks corresponding to standard Bragg reflections. The diffraction peak of 38.1° relates to (111), 44.5° relates to (200), 64.7° relates to (220), and 77.8° relates to (311) facets of the face center cubic (FCC) crystal lattice.

XRD analysis (Figure 6) revealed four important peaks present in the (20–80) 2θ range. The diffraction peaks of 38.1° relates to (111), 44.5° relates to (200), 64.7° relates to (220), and 77.8° relates to (311) facets of the face center cubic (FCC) crystal lattice; these agree with reported values for similar gold nanostructures [69]. The reported peak values also matched the planes and face-centered cubic structures of AuNPs prepared by other green syntheses methods [22,70,71,72].

#### 3.2.6. Thermogravimetric Analysis of Capped AuNPs

**Figure 7:**
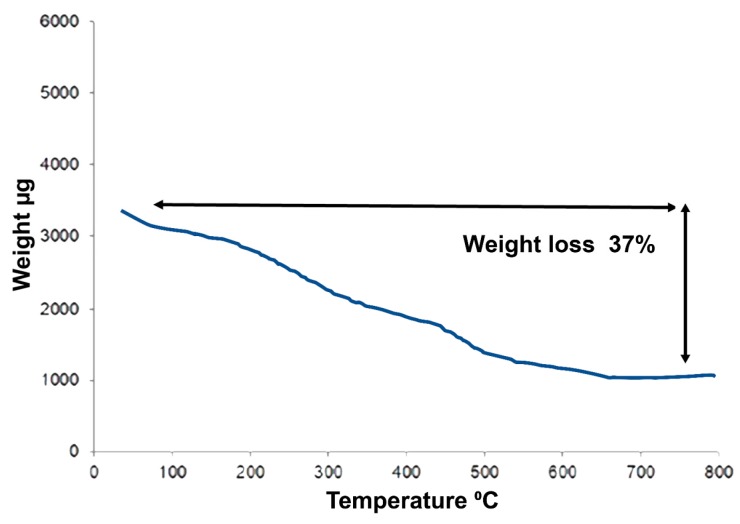
Thermogravimetric analysis of AuNPs. The curve shows the decomposition of the AuNPs coating. The freeze-dried nanoparticles weight loss was monitored against temperature increase. The total weight loss from of 37% of the starting materials suggested that the particles were coated with phenolic and other plant proteins that stabilize the particles.

TGA analysis (Figure 7) was used to determine the total amount of phytochemical residuals that capped the AuNPs ranging from phenolic compounds and small proteins that might be present in the plant extract and were adsorbed on the nanoparticles surface. Following the rigorous purification methods, impurities within the sample could be eliminated. TGA analysis showed that approximately 37% of organic components of AuNPs were degraded, suggesting that the biological ingredients from the plant extract capped the AuNPs’ surface. Furthermore, this might be related to the shift in the Raman peak range between λ_max_ 527 and 535 nm. The Raman spectra shifting is related to the chemical bond length of molecules and the nanoparticles symmetry [73,74,75]. 

#### 3.2.7. Antimicrobial Activity

##### Zone of Inhibition

Before testing antimicrobial activity, the samples were carefully characterized to eliminate any confounding variable that may affect the activity. The antifungal and antibacterial activities of AuNPs can be affected by the existence of contaminants within the sample [76]. For instance, cation contaminants interfere with antifungal activity either by inducing the hyphae form (as in calcium) or increasing the activity (as in zinc) [77,78,79]. It was essential to ensure that the AuNPs were completely pure using rigorous purification methods as described in Section 2.2.2. 

**Figure 8:**
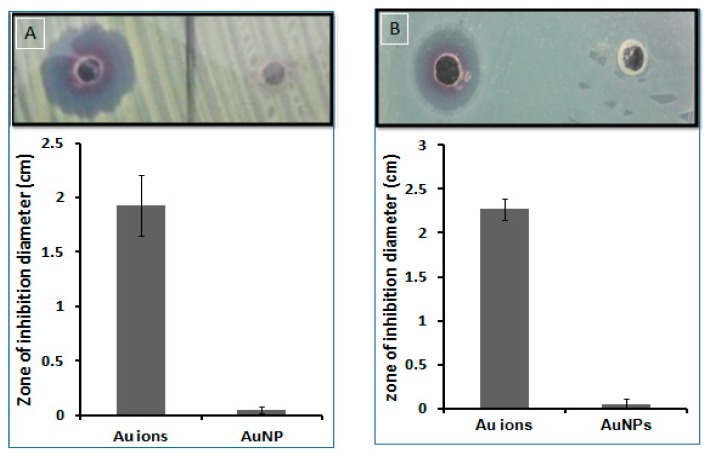
Antimicrobial activity of AuNPs and gold ions using a zone of inhibition assay. (**A**) Antifungal activity on *C. albicans* and (**B**) antibacterial activity on *E. coli*. Error bars represent the standard deviation of three independent experiments. Upper panel photographic image shows the inhibition zone of the Au^3+^ ions left and AnNPs right.

It has been demonstrated that AuNPs possess antibacterial and antifungal activities [76,80,81], whereas the antimicrobial activity is dependent on the method of synthesis, size, shape, and concentration of the generated NPs [76,81]. The antibacterial and antifungal activities of the AuNPs prepared in this work were evaluated via a zone of inhibition assay. Figure 8 shows that the AuNPs at a concentration of 5 mg/mL (as determined spectrophotometrically) did not display any activity on *E. coli*, *S. marcescens* (data not shown), or *C. albicans*, whilst equivalent aqueous free gold ions, of the reaction starting concentration, did show antimicrobial activity. 

**Figure 9:**
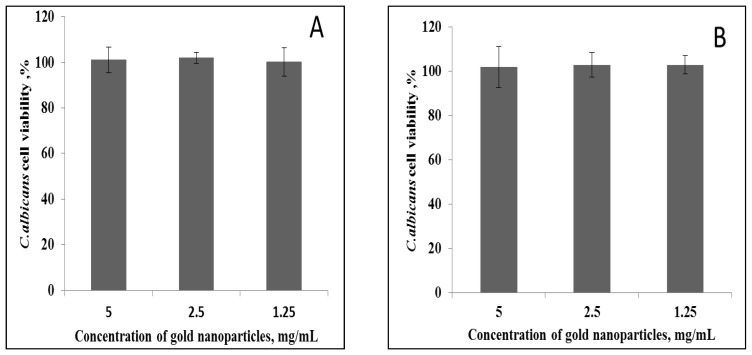
The antifungal activity of AuNPs at different concentrations using (**A**) microdilution plate assay and (**B**) plate spotting assay.

The average diameter of the zone of inhibition of *E. coli* was 22.5 mm and 0.5 mm for gold ions and AuNPs, respectively, whilst on *C. albicans* the average diameter was 11.2 mm and 0.3 mm, respectively (Figure 9). Importantly, this shows that the prepared AuNPs in this work are biocompatible for the tested organisms and thus may exhibit a low level of environmental hazard and toxicity. 

##### Microdilution and Plate Spotting

Despite DLS data showing that the average size of AuNPs is approximately 50 nm, the TEM data show that there is a decent population (only determined from the TEM micrographs and constituting roughly around 2% of the total imaged particles) of small-size, single sheeted AuNPs with an average diameter of 3 ± 0.5 nm (Figure 3, inset). These AuNPs are of the smallest size to be reported via a green synthesis route. 

According to the theory that antifungal activity is size-dependent [82,83], we expected to find activity from the small particles in the AuNP sample. The antifungal activity was evaluated by two different methods; microdilution assay and spot plating assay. The microdilution assay studied the antifungal activity within a short period of 2 h in a non-growing medium (phosphate buffer). The activity was evaluated on a limited number of cells (200 CFU) in a non-division status. AuNPs did not exhibit any antifungal activity up to 5 mg/mL as there were no significant differences in viability compared to the control (Figure 6).

In the spot plating assay, the antifungal activity was tested in a growing medium over a 24-h period, where the fungi are active and dividing. This ascertained whether growth and division are a prerequisite for AuNPs antifungal activity. These results confirmed that, up to 5 mg/mL concentration, AuNPs did not possess any antifungal activity. 

Antifungal activities are dependent on the size of NPs; the smaller the diameter, the greater the antifungal activity. For instance, 7-nm AuNPs were more potent antifungal agents than 15-nm AuNPs [84], and 25-nm particles were more effective on Candida than 30-nm particles [76,81,85]. As the AuNPs in this study are larger in size, ca. 50 nm, we propose that 50-nm NPs are too large to induce antifungal activities. Although the AuNPs in this study contained a small population of 3-nm particles, no antimicrobial activity was observed in all quantitative assays, even at 5 mg/mL gold concentration. Thus, the method of synthesis and the presence of free gold ions rather than the diameter of the AuNPs determines the activity. 

Unlike antibacterial activity, AuNPs antifungal activity has not been reported to change according to the method of synthesis, as all tested AuNPs have shown activities in a dose (concentration)-dependent manner [76]. The reported minimum inhibitory concentrations of AuNPs of Candida are varied, and none has exceeded 1 mg/mL. Herein, we report environment-friendly prepared AuNPs that are harmless to bacteria and fungi. It is essential that the purification of the AuNPs is rigorous and that all gold ions are removed from the AuNPs. The particles in this study were extensively purified by a combination of sucrose gradient and dialysis. 

## 4. Conclusions

In this work, we describe a simple, quick, and reproducible method for the environmentally friendly synthesis of AuNPs without the need for expensive reducing agents. Gold ions were chemically reduced to NPs by leaf extracts. Simple incubation of a leaf extract with aqueous gold ions at ambient temperature resulted in ‘medium monodisperse’ nanoparticles, suggesting that the plant extract acted as a strong reducing agent. This easy and simple procedure has several benefits which include cost-effectiveness, biocompatibility, and ease of scale-up production. 

The AuNPs show no antimicrobial or antifungal activity, up to concentrations of 5 mg/mL, irrespective of their size. It is proposed that antimicrobial and antifungal activity is a consequence of the presence of gold ions and not a property of the AuNPs. This opens the possibility for the use of AuNPs for drug delivery, oral or intranasal, without interfering with the human microbiota.
